# Repeated standing back extension exercise: Influence on muscle shear modulus change after lumbodorsal muscle fatigue

**DOI:** 10.3233/WOR-213452

**Published:** 2021-04-27

**Authors:** Tsuneo Kumamoto, Toshiaki Seko, Ryo Matsuda, Sayo Miura

**Affiliations:** a Department of Rehabilitation, Faculty of Health Science, Hokkaido Chitose College of Rehabilitation, Chitose, Hokkaido, Japan; b Department of Rehabilitation, Sinsapporo Neurosurgical Hospital, Sapporo, Hokkaido, Japan; c Department of Rehabilitation, Hokusei Hospital, Chitose, Hokkaido, Japan

**Keywords:** Shear wave elastography, multifidus muscles, low back pain, muscle stiffness

## Abstract

**BACKGROUND:**

In low back pain, multifidus muscle fibers reportedly exhibit increased stiffness. Low back pain was associated with lumbodorsal muscle fatigue. There is no report of using shear modulus to verify the mechanism of an immediate effect of exercise on low back pain. Here, temporary lumbodorsal muscle fatigue was created, simulating fatigue-related nonspecific low back pain.

**OBJECTIVE:**

To assess the effect of standing back extension exercise on fatigued lumbodorsal muscle based on the results of multifidus muscle elasticity measured using shear wave elastography.

**METHODS:**

Thirty-three healthy subjects were randomly divided into three groups. The subjects performed the Biering-Sorensen test as the fatigue-task of the lumbodorsal muscle before the standing back extension exercise. The fatigue-exercise group exercised five sets after completing the fatigue-task. The fatigue-non-exercise group remained standing for the same duration as the fatigue-exercise group without doing the exercise after the fatigue-task. The non-fatigue-exercise group exercised five sets of without performing the fatigue-task. As intra-group and inter-group factors, the shear modulus of the multifidus muscle was compared before and after the exercise.

**RESULTS:**

The shear modulus of the multifidus muscle after the standing back extension exercise was significantly lower in the fatigue-exercise group, and no significant decrease was observed in the fatigue-non-exercise and non-fatigue-exercise group.

**CONCLUSIONS:**

The standing back extension exercise improved the shear modulus of the fatigued multifidus muscle. Therefore, it was suggested that the change in the elasticity of fatigued muscle might lead to the prevention of low back pain caused by muscle fatigue.

## 1 Introduction

As revealed by a large-scale survey of low back pain conducted in Japan, 83%of individuals experience low back pain at least once in their lifetime, 25%had requested a leave of absence due to low back pain, and 10%had been absent from work for ≥4 consecutive days due to low back pain [1]. Therefore, there are many works related to office workers and packages of exercise training [2]. One of the causes of work-related low back pain has been prolonged flexion of the trunk [3]. In Japan, where the proportion of older adults is higher than the percentage worldwide, several people require nursing care; therefore, care workers and nurses are more needed. Because 30%of their work was done using trunk in a forward inclined posture, the prevalence of back pain was high [4] for these workers. Considering previous research, when the trunk was bending forward, the internal pressure of the multifidus muscle increases, muscle ischemia arising from increased intramuscular pressure was reportedly a cause of low back pain [5]. Also, patients with low back pain reportedly had less blood flow and oxygenation to the lumbodorsal muscles; this made them easily fatigued [6]. Muscle fatigue and muscle hemodynamics were closely linked [7]. Further, low back pain was associated with lumbodorsal muscle fatigue [6, 8, 9].

Intervention for low back pain targeting care workers and nurses comprising regular simple exercises for low back pain, such as standing back extension exercise (SBEE), was reportedly expected to im-prove and prevent low back pain caused by lumbodorsal muscle fatigue [10]. This exercise was assumedly useful for correcting the posterior displacement of intervertebral disc nucleus pulposus [11, 12]. However, musculo-physiological changes in the actual lumbar muscle group have not been discussed. Therefore, in our previous studies, lumbodorsal muscle group hemodynamics was examined in repeated trunk extension movement using near-infrared spectroscopy. Hence, improved blood flow in the area was reported, corresponding to 1.5 cm below the skin [13].

Low back pain is associated with dysfunction of the lumbar multifidus muscles [14]. In chronic low back pain, lumbar multifidus muscle fibers reportedly exhibit increased stiffness [15]. In clinical practice, lumbar multifidus muscle stiffness is often verified by palpation; however, this method is subjective and not measurable. Recently, various studies have reported using shear wave elastography (SWE) to measure muscle viscoelasticity based on shear modulus has the advantage of calculating shear modulus using the propagation speed of the shear wave generated by the transducer; this enables quantitative measurement. The shear modulus measured using SWE correlated with muscle elongation [16] as well as muscle output [17] and serves as an indicator of muscle stiffness. To examine individuals with low back pain, Chan et al. [15] compared Young’s modulus of 12 healthy subjects and 12 subjects with low back pain in the prone position, standing position, and standing with trunk flexion using strain elastography. Young’s modulus is a measure of elasticity, equal to the ratio of the stress acting on a substance to the strain produced. They reported that the value was higher for individuals with low back pain than for healthy subjects in all postures; further, the value increased with trunk flexion. Recently, various studies have reported on the reproducibility [18] and validity [19] of shear modulus, including that of the lumbar multifidus muscles in individuals with low back pain [15, 20]. The shear modulus of the multifidus muscles was high in individuals with low back pain and was associated with the level of pain [20]. Also, reduced muscular endurance was reportedly a cause of low back pain [8, 9]. Several researchers and clinicians used the Biering-Sorensen test or a modified version of the test to evaluate the endurance of the lumbodorsal muscles. In patients with low back pain, reduced posture maintenance time in the electromyographic analysis results was used as an indicator of back muscle fatigue [21–23]. In the position of the bending and Biering-Sorensen test, compared to the upright position, the intramuscular pressure of the multifidus muscles increased, and the oxygen circulation decreased [24]. Therefore, the improvement in circulation by the SBEE in previous studies may be due to the changing elasticity of the multifidus muscle. However, it is not known whether the elasticity of the multifidus muscle changes.

The purpose of this study was to assess the effect of standing back extension exercise on fatigued lumbodorsal muscle based on the results of multifidus muscle elasticity measured using shear wave elastography. Here, a temporary muscle fatigue model for the lumbodorsal muscle was created for simulating nonspecific low back pain caused by muscle fatigue.

## 2 Methods

### 2.1 Subjects

The subjects included in this laboratory study were 33 healthy adult men [age, 23.5±6.6 years; BMI, 21.6±1.5 (mean±standard deviation)]. All subjects were medical college students who volunteered for the study. Inclusion criteria were the subjects who had no history of low back pain, neck pain, spinal surgery during the previous year. Exclusion criteria were the subjects who had a history of musculoskeletal problems related to the trunk, the neck, and the posture. Further, the subjects excluded if they were unable to lift their upper body and head from prone. Subjects were provided a complete explanation of the purpose and details of the study, and written informed consent was obtained before study initiation (Ethical review board of Hokkaido Chitose College of Rehabilitation, approval number: 18004). This study described has been carried out by The Code of Ethics of the World Medical Association (Declaration of Helsinki) for experiments involving humans.

**Fig. 1:**
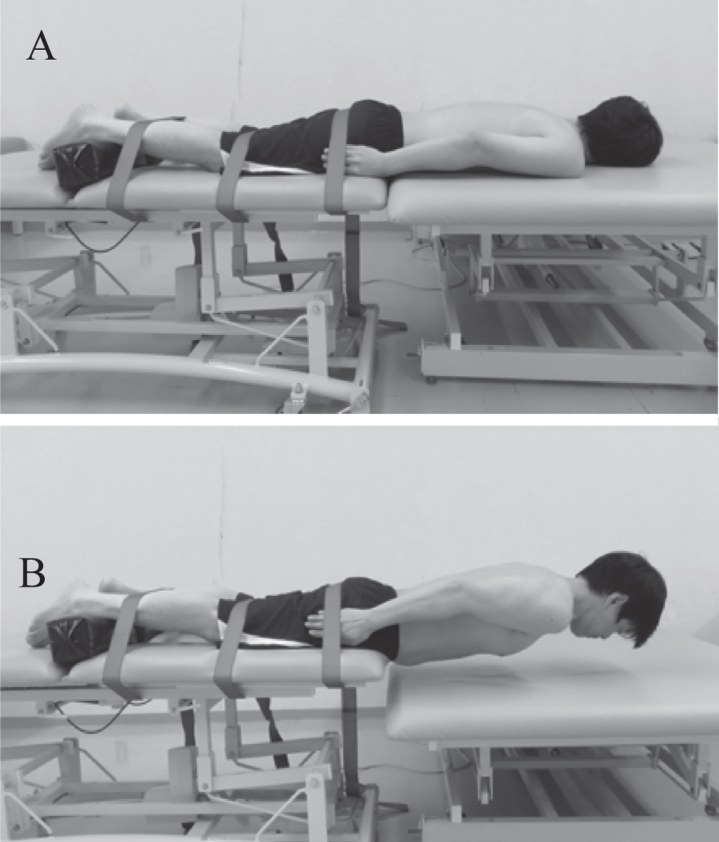
Biering-Sorensen test. A is the starting position and B is the testing posture.

We used a computer-generated random number table and randomly divided the subjects into three groups of eleven. The lumbodorsal muscle fatigue-task was to perform the Biering-Sorensen test [25] (Fig. 1). The fatigue-exercise (F-Ex) group exercised five sets of SBEE after completing the muscle fatigue-task. The fatigue-non-exercise (F-nEx) group remained standing for the same duration as the F-Ex group without doing SBEE after the muscle fatigue-task. The non-fatigue-exercise (nF-Ex) group exercised five sets without performing the muscle fatigue-task. As intra-group and inter-group factors, the shear modulus of the multifidus muscles described in the subsequent measurements was compared before and after the SBEE described in the following protocol.

### 2.2 Measurements

The shear modulus was measured using an ultrasound imaging device with SWE (AixPlorer Ver. 10.1.1.1837; SuperSonic Image, Aix-en-Provence, France) and a linear transducer (SL10-2, 2.0-10.0 MHz) in the musculoskeletal mode.

**Fig. 2:**
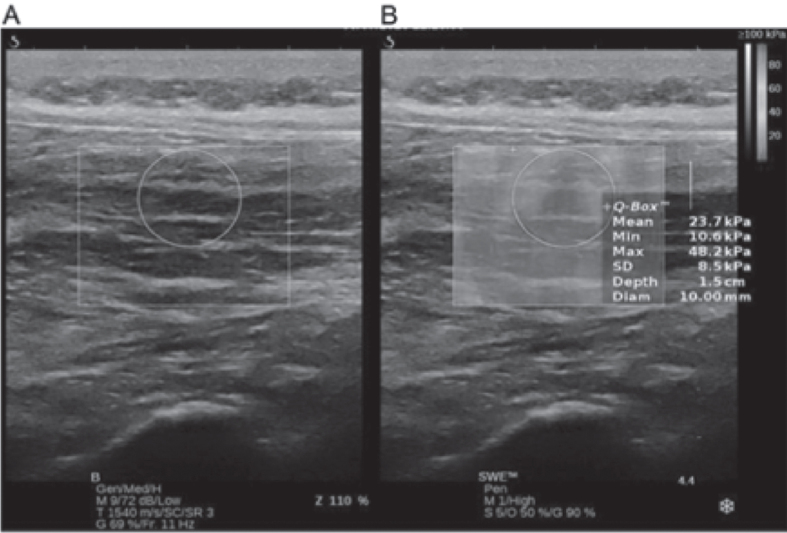
Measurement by shear wave elastography. A: B-Mode ultrasound. B: Overlays shear wave elastography to B-Mode ultrasound and measures Young’s modulus in the Q-Box^™^.

The lumbar multifidus muscles were the target muscles for shear modulus measurement. Because multifidus muscles are covered by erector spinae aponeurosis (ESA) and sheathed in the thoracolumbar fascia (TLF), muscle fibers without ESA and TLF were defined as regions of interest (ROI). Before the experiment, the iliac crest was identified by palpation, and the spinous processes of the 4th and 5th lumbar vertebrae were identified by palpation from Jacoby’s line connecting the left and right iliac crests. Thereafter, the transducer was placed transverse to the site of the spinous process of the 5th lumbar vertebra; further, in ultrasound B-mode transverse imaging, the site of the multifidus muscles was identified 2 cm lateral to the spinous process of the 5th lumbar vertebra, as per a previous study [20]. After placing the transducer over the site of the multifidus muscle belly identified via transverse imaging, the transducer was longitudinally rotated. Because the fibers of the multifidus muscles lie at a 0°–25° angle [19], the lower end of the transducer was positioned at a somewhat lateral angle; an adhesive patch was then placed on the skin at the position of the external margin of the transducer to visualize the multifidus muscle fibers via ultrasound B-mode imaging. The position of the transducer during the experiment was kept consistent with this patch as the indicator. In doing so, the depth of the multifidus muscles that could be identified was 1–3 cm, as per an earlier study [19]. When performing SWE, ultrasound B-mode and elasticity images of the ROI indicated with color mapping were viewed in dual display. Ultrasound gel was thoroughly spread on the body surface such that the pressure applied to the skin would not cause muscle deformation; operations were performed with due care, coordinating the transducer angle to visualize the multifidus muscle fibers with maximum clarity. The same tester performed all SWE operations of the multifidus muscle site using the ultrasound device system. The software (Q-Box^™^) installed in the ultrasound system was used to measure the mean shear modulus across circular ROI. Considering the depth of the multifidus muscles that could be identified, a depth of 1.5 cm from the skin was set as a baseline at the center of the circle on the Q-Box^™^; next, the mean value was calculated for measurements within the circle centered on the multifidus muscle belly, with a diameter sized to contain the maximum area of the ROI displaying clear multifidus muscle fibers (Fig. 2). These measurements were taken by a different individual who was blinded to the measurement conditions of the image data. Also, Young’s modulus has been shown for measurements obtained using the Q-Box^™^. Therefore, the values obtained were divided by three to obtain the shear modulus, as achieved in previous studies [19, 20, 26]. Regarding the reproducibility of the shear modulus, Moreau B et al. [18] reported high reproducibility at the L4-L5 level, with an intra-class correlation coefficient (ICC) of 0.92. As a pilot study, the current study confirmed the reproducibility for the elasticity of the multifidus muscles while standing at rest in healthy subjects with no history of motor disorder and central nervous system disease (*n* = 17; age, 22.2±5.0 years; height, 171.1±5.4 cm; body weight, 63.2±3.3 kg; body mass index, 25.7±8.7). Hence, the ICC (1, 1) was 0.93 (95%confidence interval, 0.82–0.97).

### 2.3 Protocol

In the F-Ex and F-nEx groups, the subjects performed the Biering-Sorensen test [25] for 60 s to fatigue the lumbodorsal muscle (Fig. 1). For this, a compact digital level gage (DI-100M, KOD, Japan) was placed between the shoulder girdle to verify that the trunk was maintained parallel with the floor, and the subjects were verbally instructed to keep their trunk horizontal when the inclination was ≥5° from horizontal.

**Fig. 3:**
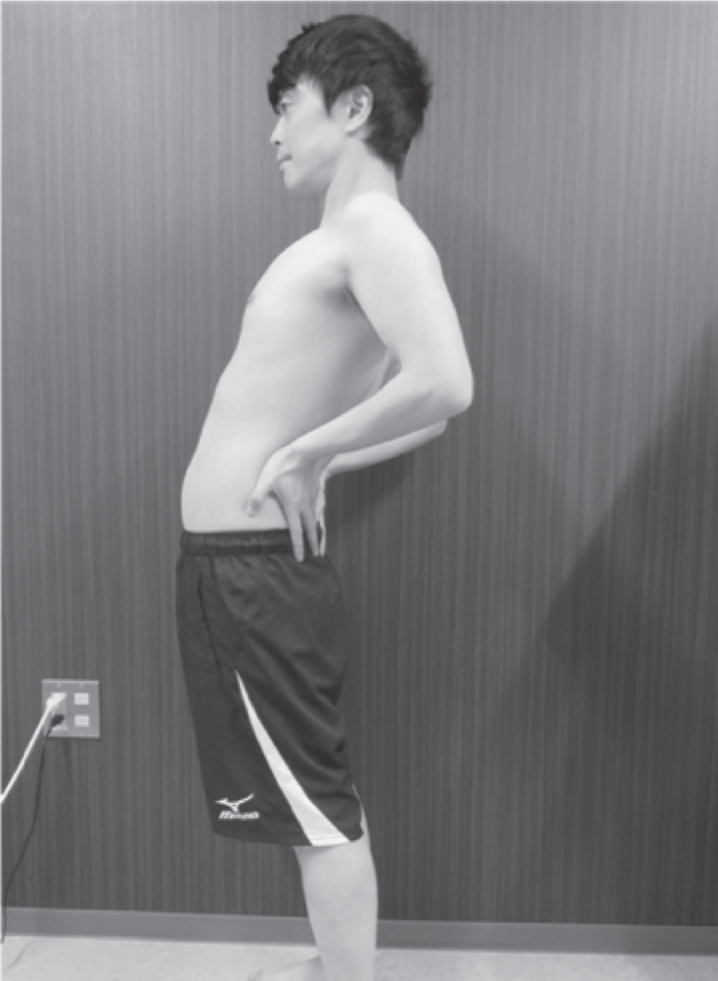
Standing back extension exercise. Extend the trunk to push the pelvis forward from the resting standing position.

Regarding the leg position, when standing, the subjects were asked to widen their stance to approximately shoulder width. Based on the results of previous studies [27, 28], using a palpation meter (PALM®; Performance Attainment Associates, St. Paul Minnesota, USA), the anterior and posterior superior iliac spines were marked such that the pelvic anteversion angle between the two points was set between 6° and 7°. With the palms of the hands placed on the posterior surfaces of the iliac crests when in a standing position, the starting standing position (pre-Ex) was defined as the 10 s after a 30 s acclimatization period. After the pre-Ex position, the subjects in the F-Ex and nF-Ex groups performed a standing back extension exercise. First, they pushed their pelvis forward from the posterior surfaces of the iliac crests with the palms of their hands while looking forward: extended their trunk as much as possible in 4 s. They maintained the maximum extended position for 10 s (Fig. 3). Next, they returned to the standing position in 4 s and sustained the standing position for 10 s (post-Ex). The F-nEx group kept the standing position for 28 s after the pre-Ex position. Each group performed five sets of this exercise (post-Ex 1–5).

### 2.4 Statistical analysis

Statistical analyses were used to compare the shear modulus for each intragroup factor, i.e., pre-Ex and post Ex1–5, and for intergroup factors of each group at maintaining the standing position. Post-corrected *p*-values used analysis of variance for split-plot design (SPANOVA; six intragroup factor levels, three intergroup factor levels), and Shaffer’s modified sequentially rejective Bonferroni test for multiple comparisons. In the analysis of variance, Greenhouse-Geisser Epsilon correction was performed, and the post-adjusted *p*-value was calculated. To compare the subject characteristics of each group, a one-way analysis of variance (ANOVA) was performed. The statistical software R was used (version 2.8.1 (2008-12-22) Copyright (C) 2008, The R Foundation for Statistical Computing) for data analyses, and *p* <  0.05 was considered statistically significant.

## 3 Results

**Fig. 4:**
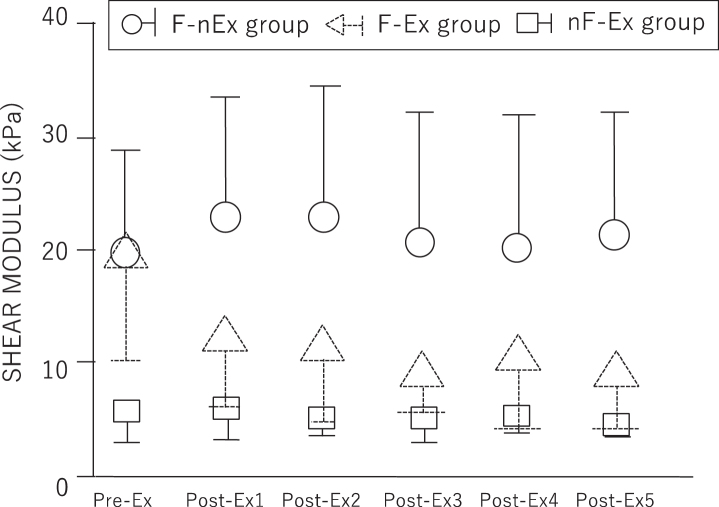
Results of shear modulus.

Comparison of individual subject characteristics by groups is presented in Table 1. There were no statistically significant difference among the three groups. No onset of adverse events caused by participation in the present study was noted in any of the subjects. SPANOVA indicated that shear modulus revealed the main effect and interaction for intergroup and intragroup factors. Results of the multiple comparisons are presented in Table 2 and Fig. 4. Multiple comparisons of intragroup factors revealed that in the F-Ex group, the shear modulus significantly decreased between pre-Ex and post-Ex3, 4, and 5 (*p* = 0.0251, 0.0066, and 0.0320, respectively). In the nF-Ex and F-nEx groups, no significant difference was observed between the pre-Ex and post-Ex shear modulus. Multiple comparisons of intergroup factors revealed a significant difference in the pre-Ex results between the F-Ex and nF-Ex groups as well as between the F-nEx and nF-Ex groups (*p* = 0.0005 and 0.0005, respectively). Further, no significant difference was observed between the F-Ex and F-nEx groups (*p* = 0.9285). In the Post-Ex1 results, a significant difference was observed between the F-Ex and F-nEx groups as well as between the nF-Ex and F-nEx groups (*p* = 0.0022 and <  0.0001, respectively). In the post-Ex2 results, a significant difference was observed between the F-Ex and F-nEx groups as well as between the nF-Ex and F-nEx groups (*p* = 0.0018 and <  0.0001, respectively). In the post-Ex3 results, a significant difference was observed between the F-Ex and F-nEx groups as well as between the nF-Ex and F-nEx groups (*p* = 0.0006 and 0.0001, respectively). In the post-Ex4 results, a significant difference was observed between the F-Ex and F-nEx groups as well as between the nF-Ex and F-nEx groups (*p* = 0.0042 and 0.0002, respectively). In the post-Ex5 results, a significant difference was observed between the F-Ex and F-nEx groups as well as between the nF-Ex and F-nEx group (*p* = 0.0003 and <  0.0001, respectively). Further, in the Post-Ex1, 2, 3, 4, and 5 results, no significant difference was observed between the F-Ex and nF-Ex groups.

**Table 1:** Characteristics of subjects in the three groups

	F-Ex (*n* = 11)^a^	nF-Ex (*n* = 11)^b^	F-nEx (*n* = 11)^c^	*P*-value
Age (years)	26.8 (8.6)	21.2 (3.7)	22.5 (5.6)	0.1269
Height (cm)	168.8 (5.9)	167.4 (4.0)	171.6 (4.4)	0.2412
Weight (kg)	61.5 (6.4)	60.5 (6.1)	63.5 (3.1)	0.1269
BMI^d^	21.5 (1.4)	21.6 (2.1)	21.6 (0.8)	0.9959

Values of each group are presented as an average (standard deviation). ^a^F-Ex: Group performed five sets of standing back extension exercise after performing the task to fatigue the lumbodorsal muscles. ^b^nF-Ex: Group performed five sets of standing back extension exercise without performing the task to fatigue the lumbodorsal muscles. ^c^F-nEx: Group remained standing for the same duration as the F-Ex group without performing standing back extension exercise after the task to fatigue the lumbodorsal muscles. ^d^BMI: body mass index.

**Table 2:** Result of shear modulus [kPa, mean (standard deviation)]

	pre-Ex^d^	post-Ex1^e^	post-Ex2^e^	post-Ex3^e^	post-Ex4^e^	post-Ex5^e^
F-Ex^a^	19.3 (9.1)^f^	12.2 (6.1)	11.5 (6.7)	9.0 (3.2)^f^	10.3 (6.0)^f^	8.9 (4.7)^f^
nF-Ex^b^	5.7 (2.9)^g^	6.1 (3.0)	5.3 (1.7)	5.4 (2.5)	5.3 (1.4)	4.7 (1.1)
F-nEx^c^	19.6 (9.1)	22.8 (11.6)^h^	22.8 (11.6)^h^	20.6 (11.6)^h^	20.3 (11.5)^h^	21.2 (11.2)^h^

^a^F-Ex: Group performed five sets of standing back extension exercise after performing the task to fatigue the lumbodorsal muscles. ^b^nF-Ex: Group performed five sets of standing back extension exercise without performing the task to fatigue the lumbodorsal muscles. ^c^F-nEx: Group remained standing for the same duration as the F-Ex group without performing standing back extension exercise after the task to fatigue the lumbodorsal muscles. ^d^pre-Ex: Resting standing position before exercise. ^e^post-Ex1–5: Standing position after standing back extension exercise 1–5 times. ^f^Intra-factor: *p* <  0.05 (pre v.s. post3, 4, 5). ^g^Inter-factor: *p* <  0.05 (F-Ex, F-nEx v.s. nF-Ex). ^h^Inter-factor: *p* <  0.05 (F-nEx v.s. F-Ex, nF-Ex).

## 4 Discussion

The idea that low back pain is not a biological impairment or anatomical abnormality, but a symptom of biopsychosocial pain syndrome is now being established. Ergonomic and psychosocial considerations are reportedly risk factors for newly developed low back pain that hinders work [10]. Further, low back pain includes specific types, such as herniated disc and spondylolysis, as well as nonspecific pain, with inconsistent imaging and clinical findings. Nonspecific low back pain reportedly accounts for 85%of low back pain, with approximately 70%being primarily caused by localized low back pain. Nonspecific low back pain included intervertebral joint pain in 27%, myofascial pain in 22%, intervertebral disc-induced pain in 16%, and sacroiliac joint pain in 7%, which was mainly caused by structures surrounding the lumbar spine [29]. Therefore, it was highly likely that low back pain indicated a problem localized in the lower back. However, the existence of many different possible causes low back pain. The biopsychosocial context causes various changes in the body. Thus, it indicates that to examine the effects, the factor in research should limit as much as possible.

Creze et al. [19] examined the shear modulus of the multifidus muscles *in vivo* in seven fresh cadavers and *ex vivo* in 16 healthy subjects. No significant difference was obtained, thus indicating that shear modulus measurement by SWE from the body surface is valid. Furthermore, Moreau et al. [18] used SWE to measure the shear modulus of the multifidus muscles in 10 healthy subjects at rest in the prone position and passive stretching position on a massage chair; they reported that the reproducibility of the shear modulus values was high in both positions. Masaki et al. [20] compared shear modulus of the erector spinae and multifidus muscles measured in the prone position using SWE in 23 young-to-middle aged healthy subjects and nine health professionals with low back pain. They found that, compared with healthy subjects, individuals with low back pain had a higher shear modulus of the multifidus muscles. Upon performing multiple regression analysis, including other parameters, such as muscle thickness and spinal column alignment, they observed a relationship between the shear modulus of the multifidus muscles and low back pain. Konno et al. [5] measured intramuscular pressure via a transducer inserted at the level of the L5 vertebra, 2–3 cm lateral to the spinous process; based on the results, they reported that bending forward while standing applies large force on the multifidus muscles and increases intramuscular pressure. For care workers and nurses who work in a bending forward posture, this considerably reduces the amount of muscular blood flow, thereby inducing muscular low back pain. Here, the shear modulus of the multifidus muscles was higher when the muscles were fatigued compared with when they were not; this indicates that continuous contraction of the lumbodorsal muscles exerts continuous pressure on the capillaries in the muscle tissue. This continuous pressure exacerbates hemodynamics, thus causing ischemic low back pain. In the group that performed SBEE after fatiguing the lumbodorsal muscles, the shear modulus was lower than before the SBEE. In our previous studies [13, 30], changes in hemodynamics of the lumbar muscle group were assessed after the performance of this exercise. The result showed that improved blood flow in the multifidus muscles. Based on McKenzie’s theory [11], the SBEE may alleviate pain caused by posterior displacement of the intervertebral disc nucleus pulposus. Furthermore, we believe that the results of the present study, the SBEE has the possibility of dilating the capillaries and hemodynamics by improving the muscle elasticity. In contrast, SBEE resulted in no change in the shear modulus in the group without fatigued lumbodorsal muscles. We believe this suggests that if SBEE does not change symptoms, it may be due to factors other than fatigue as an indicator of the clinical subgrouping of chronic low back pain.

The present study has some limitations. Even with muscle fatigue loading, the study included only healthy subjects; therefore, one cannot confirm that the subsequent hemodynamic-improving ability is impaired. Hemodynamics reportedly improves more slowly in individuals with low back pain than in healthy adults after maintaining a trunk extension posture [31]. Further analysis is warranted for subjects with myofascial low back pain caused by ischemia. The stimulation threshold of the intervertebral joint by SBEE possibly differs from that in individuals with low back pain. In the present study, the shear modulus was higher in the group with multifidus muscle fatigue than in the group without multifidus muscle fatigue. Therefore, an investigation of change overtime may be required. In doing so, we would like to examine changes in the shear modulus as well as in hemodynamics. We believe that the results of the present study are valuable for data comparison in individuals with low back pain with impaired ability to improve hemodynamics of the lumbodorsal muscles as well as those with problems regarding the stimulation threshold of the intervertebral joints.

## 5 Conclusion

In the present study, for simulating fatigue-related low back pain, a temporary lumbodorsal muscle fatigue model was created. After the fatigue of the lumbodorsal muscle, the result of performing the repeated SBEE showed improvement in the elastic modulus of the fatigued multifidus muscle. Therefore, the results obtained in the present study suggest that performing the SBEE occurred the change in the elasticity of fatigued lumbodorsal muscle might lead to the prevention of muscle fatigue-related low back pain.

This exercise is simpler than conventional training, which is beneficial because it can be done at work. The idea that low back pain is not only a biological impairment or anatomical abnormality but also a biopsychosocial pain syndrome is now being established. Since this study was for healthy subjects, it is necessary to verify fatigue-related ischemic low back pain, including other psychosocial factors. We hope to explore the changing elasticity of multifidus muscle in future research with the subjects of nurses and care workers with muscle fatigue-related low back pain. Further, it is required to synchronize the measurement of hemodynamics and elasticity and to verify long-term effects.
